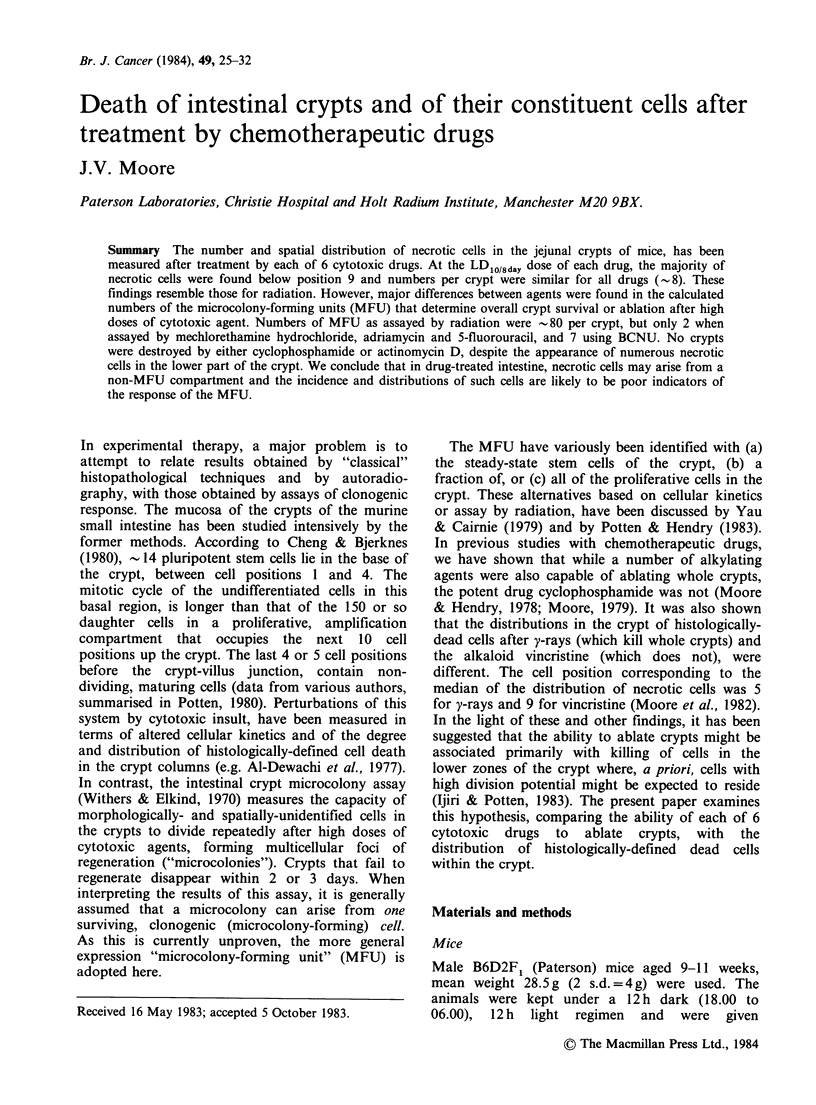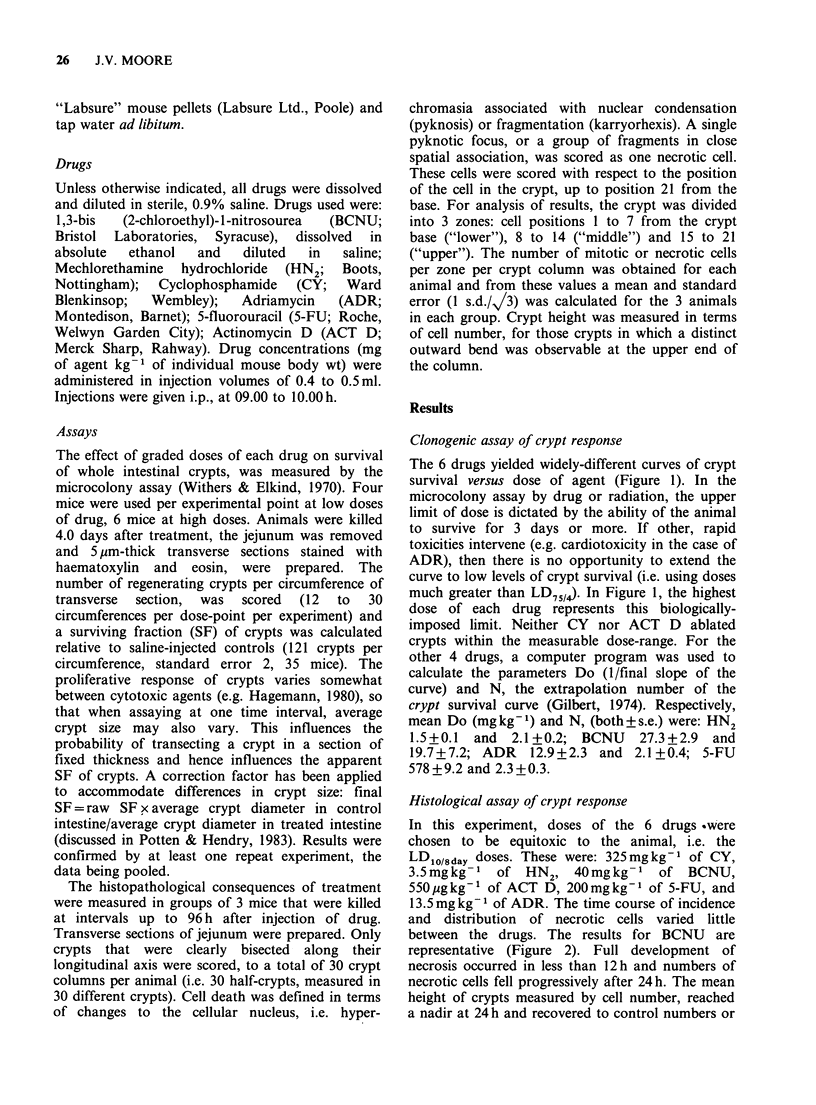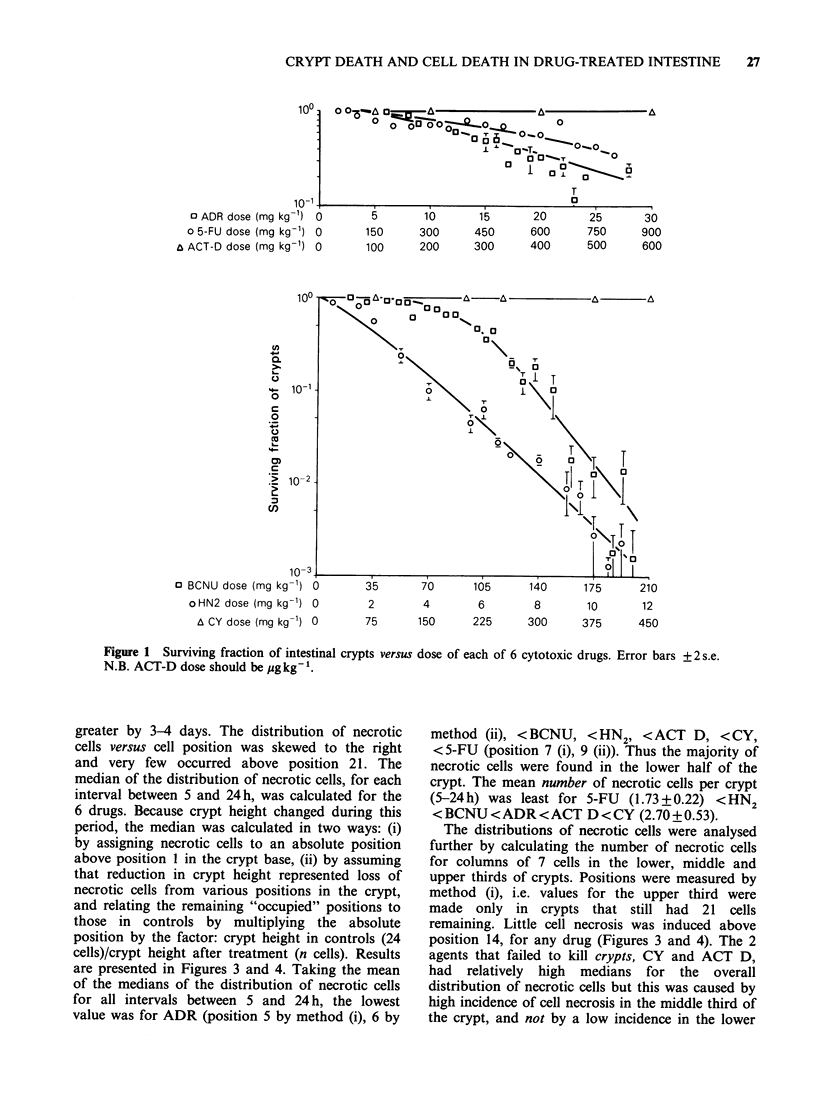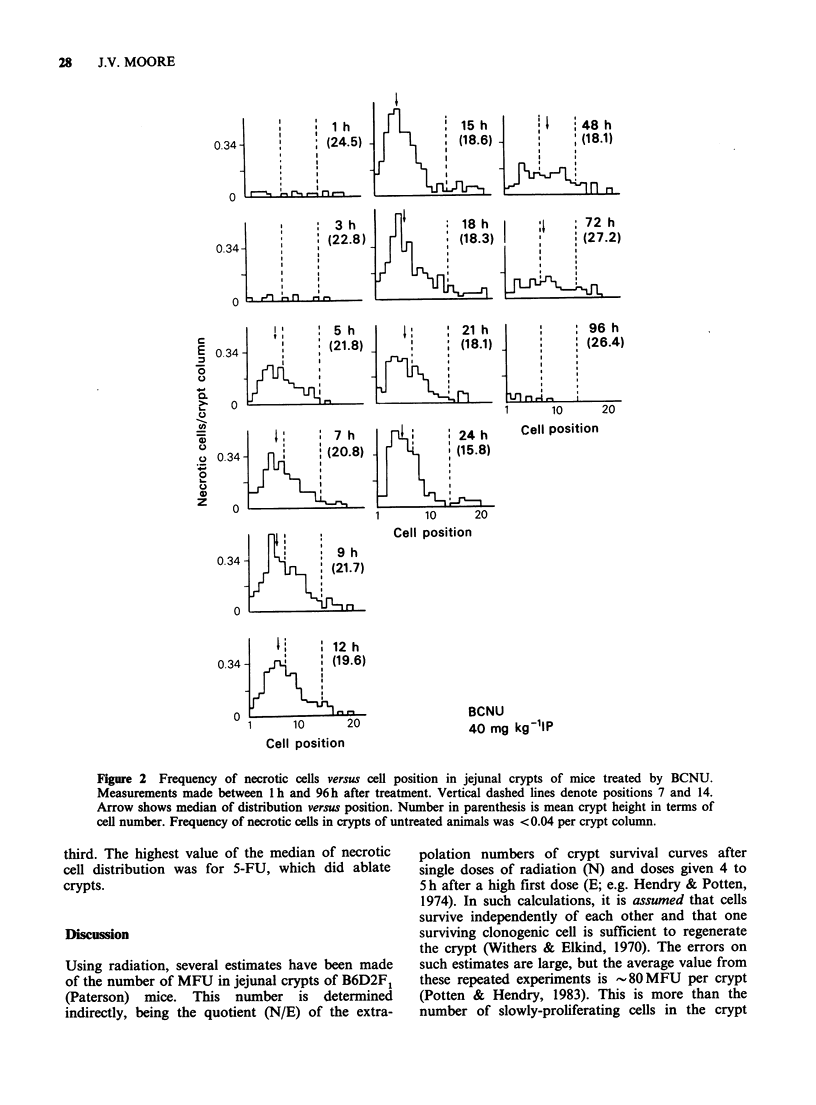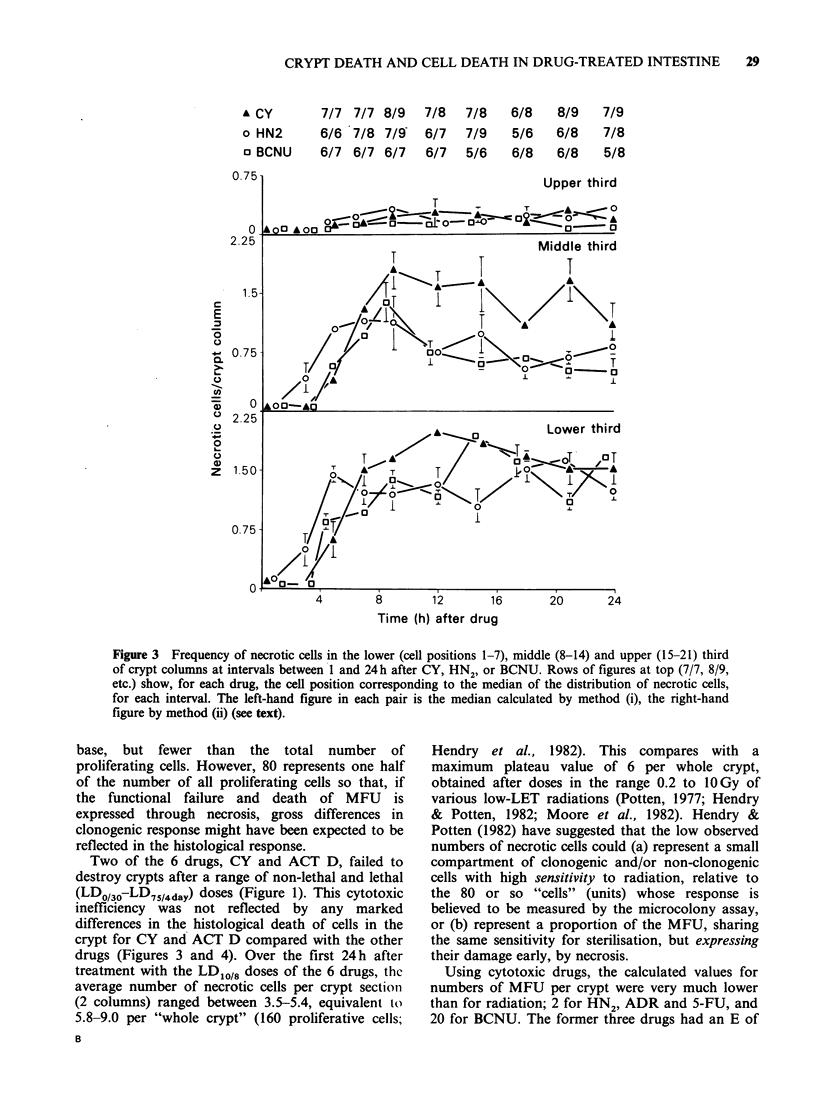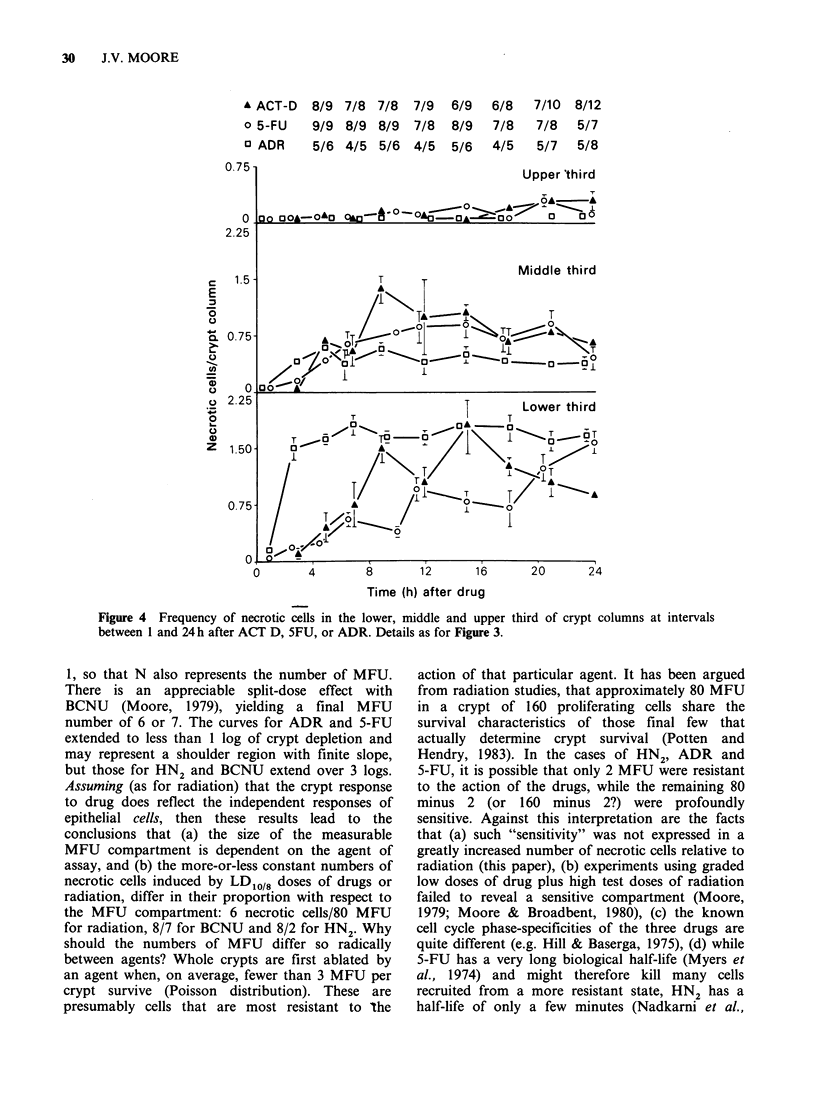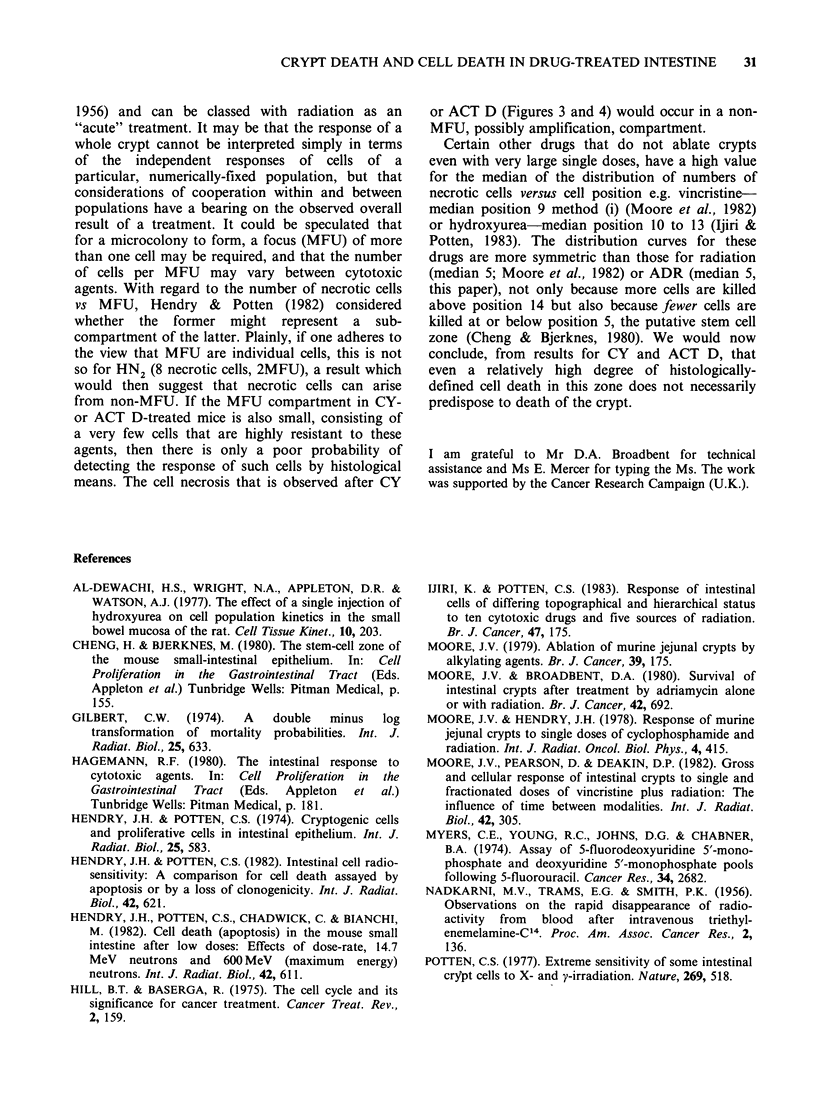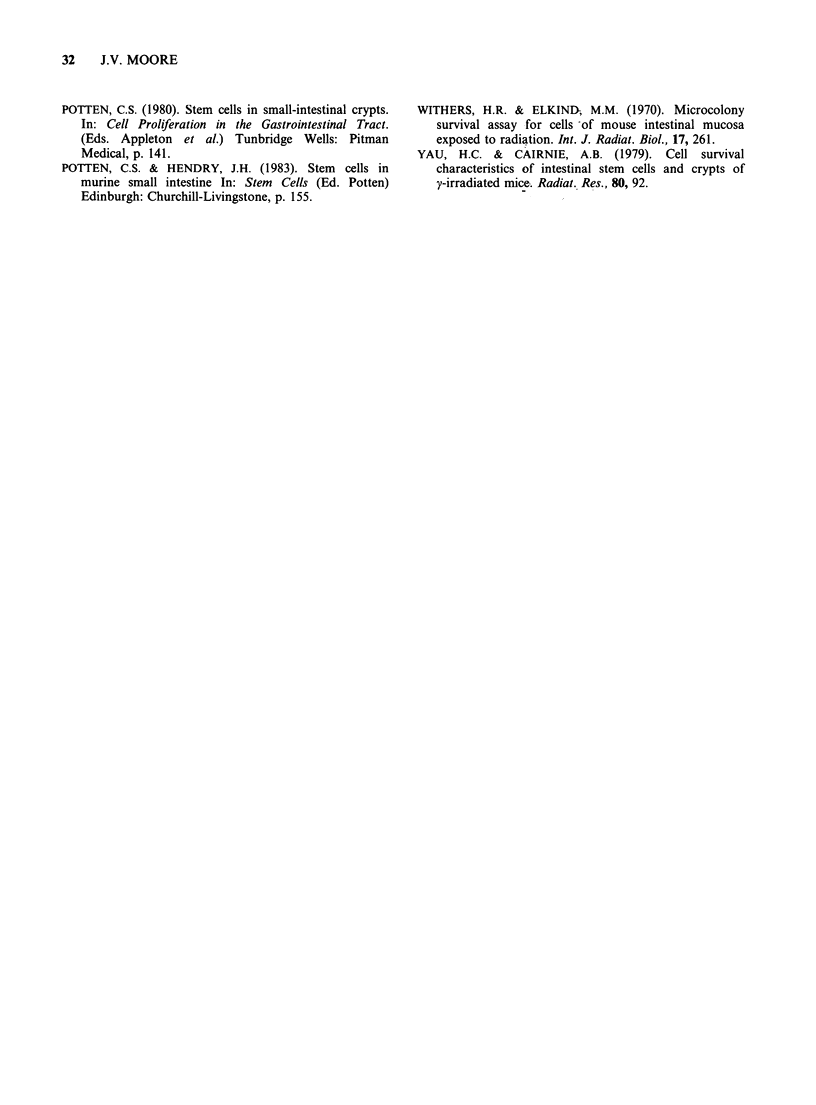# Death of intestinal crypts and of their constituent cells after treatment by chemotherapeutic drugs.

**DOI:** 10.1038/bjc.1984.5

**Published:** 1984-01

**Authors:** J. V. Moore

## Abstract

The number and spatial distribution of necrotic cells in the jejunal crypts of mice, has been measured after treatment by each of 6 cytotoxic drugs. At the LD10/8 day dose of each drug, the majority of necrotic cells were found below position 9 and numbers per crypt were similar for all drugs (approximately 8). These findings resemble those for radiation. However, major differences between agents were found in the calculated numbers of the microcolony-forming units (MFU) that determine overall crypt survival or ablation after high doses of cytotoxic agent. Numbers of MFU as assayed by radiation were approximately 80 per crypt, but only 2 when assayed by mechlorethamine hydrochloride, adriamycin and 5-fluorouracil, and 7 using BCNU. No crypts were destroyed by either cyclophosphamide or actinomycin D, despite the appearance of numerous necrotic cells in the lower part of the crypt. We conclude that in drug-treated intestine, necrotic cells may arise from a non-MFU compartment and the incidence and distributions of such cells are likely to be poor indicators of the response of the MFU.


*Br. J. Cancer* (1984), **49**, 25–32

### Death of intestinal crypts and of their constituent cells after treatment by chemotherapeutic drugs

J.V. Moore

*Paterson Laboratories, Christie Hospital and Holt Radium Institute, Manchester M20 9BX.*

**Summary** The number and spatial distribution of necrotic cells in the jejunal crypts of mice, has been measured after treatment by each of 6 cytotoxic drugs. At the $LD_{10/8day}$ dose of each drug, the majority of necrotic cells were found below position 9 and numbers per crypt were similar for all drugs (~8). These findings resemble those for radiation. However, major differences between agents were found in the calculated numbers of the microcolony-forming units (MFU) that determine overall crypt survival or ablation after high doses of cytotoxic agent. Numbers of MFU as assayed by radiation were ~80 per crypt, but only 2 when assayed by mechlorethamine hydrochloride, adriamycin and 5-fluorouracil, and 7 using BCNU. No crypts were destroyed by either cyclophosphamide or actinomycin D, despite the appearance of numerous necrotic cells in the lower part of the crypt. We conclude that in drug-treated intestine, necrotic cells may arise from a non-MFU compartment and the incidence and distributions of such cells are likely to be poor indicators of the response of the MFU.

In experimental therapy, a major problem is to attempt to relate results obtained by "classical" histopathological techniques and by autoradiography, with those obtained by assays of clonogenic response. The mucosa of the crypts of the murine small intestine has been studied intensively by the former methods. According to Cheng & Bjerknes (1980), ~14 pluripotent stem cells lie in the base of the crypt, between cell positions 1 and 4. The mitotic cycle of the undifferentiated cells in this basal region, is longer than that of the 150 or so daughter cells in a proliferative, amplification compartment that occupies the next 10 cell positions up the crypt. The last 4 or 5 cell positions before the crypt-villus junction, contain non-dividing, maturing cells (data from various authors, summarised in Potten, 1980). Perturbations of this system by cytotoxic insult, have been measured in terms of altered cellular kinetics and of the degree and distribution of histologically-defined cell death in the crypt columns (e.g. Al-Dewachi *et al.*, 1977). In contrast, the intestinal crypt microcolony assay (Withers & Elkind, 1970) measures the capacity of morphologically- and spatially-unidentified cells in the crypts to divide repeatedly after high doses of cytotoxic agents, forming multicellular foci of regeneration ("microcolonies"). Crypts that fail to regenerate disappear within 2 or 3 days. When interpreting the results of this assay, it is generally assumed that a microcolony can arise from *one* surviving, clonogenic (microcolony-forming) *cell.* As this is currently unproven, the more general expression "microcolony-forming unit" (MFU) is adopted here.

Received 16 May 1983; accepted 5 October 1983.

The MFU have variously been identified with (a) the steady-state stem cells of the crypt, (b) a fraction of, or (c) all of the proliferative cells in the crypt. These alternatives based on cellular kinetics or assay by radiation, have been discussed by Yau & Cairnie (1979) and by Potten & Hendry (1983). In previous studies with chemotherapeutic drugs, we have shown that while a number of alkylating agents were also capable of ablating whole crypts, the potent drug cyclophosphamide was not (Moore & Hendry, 1978; Moore, 1979). It was also shown that the distributions in the crypt of histologically-dead cells after γ-rays (which kill whole crypts) and the alkaloid vincristine (which does not), were different. The cell position corresponding to the median of the distribution of necrotic cells was 5 for γ-rays and 9 for vincristine (Moore *et al.*, 1982). In the light of these and other findings, it has been suggested that the ability to ablate crypts might be associated primarily with killing of cells in the lower zones of the crypt where, *a priori,* cells with high division potential might be expected to reside (Ijiri & Potten, 1983). The present paper examines this hypothesis, comparing the ability of each of 6 cytotoxic drugs to ablate crypts, with the distribution of histologically-defined dead cells within the crypt.

### Materials and methods

### *Mice*

Male $B6D2F_1$ (Paterson) mice aged 9–11 weeks, mean weight 28.5 g (2 s.d. = 4 g) were used. The animals were kept under a 12 h dark (18.00 to 06.00), 12 h light regimen and were given

© The Macmillan Press Ltd., 1984

"Labsure" mouse pellets (Labsure Ltd., Poole) and tap water *ad libitum*.

*Drugs*

Unless otherwise indicated, all drugs were dissolved and diluted in sterile, 0.9% saline. Drugs used were: 1,3-bis (2-chloroethyl)-1-nitrosourea (BCNU; Bristol Laboratories, Syracuse), dissolved in absolute ethanol and diluted in saline; Mechlorethamine hydrochloride ($HN_2$; Boots, Nottingham); Cyclophosphamide (CY; Ward Blenkinsop; Wembley); Adriamycin (ADR; Montedison, Barnet); 5-fluorouracil (5-FU; Roche, Welwyn Garden City); Actinomycin D (ACT D; Merck Sharp, Rahway). Drug concentrations (mg of agent $kg^{-1}$ of individual mouse body wt) were administered in injection volumes of 0.4 to 0.5 ml. Injections were given i.p., at 09.00 to 10.00 h.

*Assays*

The effect of graded doses of each drug on survival of whole intestinal crypts, was measured by the microcolony assay (Withers & Elkind, 1970). Four mice were used per experimental point at low doses of drug, 6 mice at high doses. Animals were killed 4.0 days after treatment, the jejunum was removed and 5 $\mu$m-thick transverse sections stained with haematoxylin and eosin, were prepared. The number of regenerating crypts per circumference of transverse section, was scored (12 to 30 circumferences per dose-point per experiment) and a surviving fraction (SF) of crypts was calculated relative to saline-injected controls (121 crypts per circumference, standard error 2, 35 mice). The proliferative response of crypts varies somewhat between cytotoxic agents (e.g. Hagemann, 1980), so that when assaying at one time interval, average crypt size may also vary. This influences the probability of transecting a crypt in a section of fixed thickness and hence influences the apparent SF of crypts. A correction factor has been applied to accommodate differences in crypt size: final SF = raw SF $\times$ average crypt diameter in control intestine/average crypt diameter in treated intestine (discussed in Potten & Hendry, 1983). Results were confirmed by at least one repeat experiment, the data being pooled.

The histopathological consequences of treatment were measured in groups of 3 mice that were killed at intervals up to 96 h after injection of drug. Transverse sections of jejunum were prepared. Only crypts that were clearly bisected along their longitudinal axis were scored, to a total of 30 crypt columns per animal (i.e. 30 half-crypts, measured in 30 different crypts). Cell death was defined in terms of changes to the cellular nucleus, i.e. hyperchromasia associated with nuclear condensation (pyknosis) or fragmentation (karryorhexis). A single pyknotic focus, or a group of fragments in close spatial association, was scored as one necrotic cell. These cells were scored with respect to the position of the cell in the crypt, up to position 21 from the base. For analysis of results, the crypt was divided into 3 zones: cell positions 1 to 7 from the crypt base ("lower"), 8 to 14 ("middle") and 15 to 21 ("upper"). The number of mitotic or necrotic cells per zone per crypt column was obtained for each animal and from these values a mean and standard error (1 s.d./$\sqrt{3}$) was calculated for the 3 animals in each group. Crypt height was measured in terms of cell number, for those crypts in which a distinct outward bend was observable at the upper end of the column.

### Results

*Clonogenic assay of crypt response*

The 6 drugs yielded widely-different curves of crypt survival *versus* dose of agent (Figure 1). In the microcolony assay by drug or radiation, the upper limit of dose is dictated by the ability of the animal to survive for 3 days or more. If other, rapid toxicities intervene (e.g. cardiotoxicity in the case of ADR), then there is no opportunity to extend the curve to low levels of crypt survival (i.e. using doses much greater than $LD_{75/4}$). In Figure 1, the highest dose of each drug represents this biologically-imposed limit. Neither CY nor ACT D ablated crypts within the measurable dose-range. For the other 4 drugs, a computer program was used to calculate the parameters Do (1/final slope of the curve) and N, the extrapolation number of the *crypt* survival curve (Gilbert, 1974). Respectively, mean Do (mg $kg^{-1}$) and N, (both $\pm$ s.e.) were: $HN_2$ 1.5 $\pm$ 0.1 and 2.1 $\pm$ 0.2; BCNU 27.3 $\pm$ 2.9 and 19.7 $\pm$ 7.2; ADR 12.9 $\pm$ 2.3 and 2.1 $\pm$ 0.4; 5-FU 578 $\pm$ 9.2 and 2.3 $\pm$ 0.3.

*Histological assay of crypt response*

In this experiment, doses of the 6 drugs were chosen to be equitoxic to the animal, i.e. the $LD_{10/8day}$ doses. These were: 325 mg $kg^{-1}$ of CY, 3.5 mg $kg^{-1}$ of $HN_2$, 40 mg $kg^{-1}$ of BCNU, 550 $\mu$g $kg^{-1}$ of ACT D, 200 mg $kg^{-1}$ of 5-FU, and 13.5 mg $kg^{-1}$ of ADR. The time course of incidence and distribution of necrotic cells varied little between the drugs. The results for BCNU are representative (Figure 2). Full development of necrosis occurred in less than 12 h and numbers of necrotic cells fell progressively after 24 h. The mean height of crypts measured by cell number, reached a nadir at 24 h and recovered to control numbers or

**Figure 1** Surviving fraction of intestinal crypts *versus* dose of each of 6 cytotoxic drugs. Error bars $\pm 2$ s.e. N.B. ACT-D dose should be $\mu g\,kg^{-1}$.

greater by 3–4 days. The distribution of necrotic cells *versus* cell position was skewed to the right and very few occurred above position 21. The median of the distribution of necrotic cells, for each interval between 5 and 24 h, was calculated for the 6 drugs. Because crypt height changed during this period, the median was calculated in two ways: (i) by assigning necrotic cells to an absolute position above position 1 in the crypt base, (ii) by assuming that reduction in crypt height represented loss of necrotic cells from various positions in the crypt, and relating the remaining "occupied" positions to those in controls by multiplying the absolute position by the factor: crypt height in controls (24 cells)/crypt height after treatment (*n* cells). Results are presented in Figures 3 and 4. Taking the mean of the medians of the distribution of necrotic cells for all intervals between 5 and 24 h, the lowest value was for ADR (position 5 by method (i), 6 by method (ii), <BCNU, <$HN_2$, <ACT D, <CY, <5-FU (position 7 (i), 9 (ii)). Thus the majority of necrotic cells were found in the lower half of the crypt. The mean *number* of necrotic cells per crypt (5–24 h) was least for 5-FU ($1.73 \pm 0.22$) <$HN_2$ <BCNU<ADR<ACT D<CY ($2.70 \pm 0.53$).

The distributions of necrotic cells were analysed further by calculating the number of necrotic cells for columns of 7 cells in the lower, middle and upper thirds of crypts. Positions were measured by method (i), i.e. values for the upper third were made only in crypts that still had 21 cells remaining. Little cell necrosis was induced above position 14, for any drug (Figures 3 and 4). The 2 agents that failed to kill *crypts*, CY and ACT D, had relatively high medians for the overall distribution of necrotic cells but this was caused by high incidence of cell necrosis in the middle third of the crypt, and *not* by a low incidence in the lower

**Figure 2** Frequency of necrotic cells *versus* cell position in jejunal crypts of mice treated by BCNU. Measurements made between 1 h and 96 h after treatment. Vertical dashed lines denote positions 7 and 14. Arrow shows median of distribution *versus* position. Number in parenthesis is mean crypt height in terms of cell number. Frequency of necrotic cells in crypts of untreated animals was $<0.04$ per crypt column.

third. The highest value of the median of necrotic cell distribution was for 5-FU, which did ablate crypts.

### Discussion

Using radiation, several estimates have been made of the number of MFU in jejunal crypts of $B6D2F_1$ (Paterson) mice. This number is determined indirectly, being the quotient (N/E) of the extrapolation numbers of crypt survival curves after single doses of radiation (N) and doses given 4 to 5 h after a high first dose (E; e.g. Hendry & Potten, 1974). In such calculations, it is *assumed* that cells survive independently of each other and that one surviving clonogenic cell is sufficient to regenerate the crypt (Withers & Elkind, 1970). The errors on such estimates are large, but the average value from these repeated experiments is ~80 MFU per crypt (Potten & Hendry, 1983). This is more than the number of slowly-proliferating cells in the crypt

| | | | | | | | | |
|---|---|---|---|---|---|---|---|---|
| ▲ CY | 7/7 | 7/7 | 8/9 | 7/8 | 7/8 | 6/8 | 8/9 | 7/9 |
| o HN2 | 6/6 | 7/8 | 7/9 | 6/7 | 7/9 | 5/6 | 6/8 | 7/8 |
| □ BCNU | 6/7 | 6/7 | 6/7 | 6/7 | 5/6 | 6/8 | 6/8 | 5/8 |

**Figure 3** Frequency of necrotic cells in the lower (cell positions 1–7), middle (8–14) and upper (15–21) third of crypt columns at intervals between 1 and 24 h after CY, $HN_2$, or BCNU. Rows of figures at top (7/7, 8/9, etc.) show, for each drug, the cell position corresponding to the median of the distribution of necrotic cells, for each interval. The left-hand figure in each pair is the median calculated by method (i), the right-hand figure by method (ii) (see text).

base, but fewer than the total number of proliferating cells. However, 80 represents one half of the number of all proliferating cells so that, if the functional failure and death of MFU is expressed through necrosis, gross differences in clonogenic response might have been expected to be reflected in the histological response.

Two of the 6 drugs, CY and ACT D, failed to destroy crypts after a range of non-lethal and lethal ($LD_{0/30}$–$LD_{75/4day}$) doses (Figure 1). This cytotoxic inefficiency was not reflected by any marked differences in the histological death of cells in the crypt for CY and ACT D compared with the other drugs (Figures 3 and 4). Over the first 24 h after treatment with the $LD_{10/8}$ doses of the 6 drugs, the average number of necrotic cells per crypt section (2 columns) ranged between 3.5–5.4, equivalent to 5.8–9.0 per "whole crypt" (160 proliferative cells; Hendry *et al.*, 1982). This compares with a maximum plateau value of 6 per whole crypt, obtained after doses in the range 0.2 to 10 Gy of various low-LET radiations (Potten, 1977; Hendry & Potten, 1982; Moore *et al.*, 1982). Hendry & Potten (1982) have suggested that the low observed numbers of necrotic cells could (a) represent a small compartment of clonogenic and/or non-clonogenic cells with high *sensitivity* to radiation, relative to the 80 or so "cells" (units) whose response is believed to be measured by the microcolony assay, or (b) represent a proportion of the MFU, sharing the same sensitivity for sterilisation, but *expressing* their damage early, by necrosis.

Using cytotoxic drugs, the calculated values for numbers of MFU per crypt were very much lower than for radiation; 2 for $HN_2$, ADR and 5-FU, and 20 for BCNU. The former three drugs had an E of

**Figure 4** Frequency of necrotic cells in the lower, middle and upper third of crypt columns at intervals between 1 and 24 h after ACT D, 5FU, or ADR. Details as for **Figure 3**.

1, so that N also represents the number of MFU. There is an appreciable split-dose effect with BCNU (Moore, 1979), yielding a final MFU number of 6 or 7. The curves for ADR and 5-FU extended to less than 1 log of crypt depletion and may represent a shoulder region with finite slope, but those for $HN_2$ and BCNU extend over 3 logs. *Assuming* (as for radiation) that the crypt response to drug does reflect the independent responses of epithelial *cells*, then these results lead to the conclusions that (a) the size of the measurable MFU compartment is dependent on the agent of assay, and (b) the more-or-less constant numbers of necrotic cells induced by $LD_{10/8}$ doses of drugs or radiation, differ in their proportion with respect to the MFU compartment: 6 necrotic cells/80 MFU for radiation, 8/7 for BCNU and 8/2 for $HN_2$. Why should the numbers of MFU differ so radically between agents? Whole crypts are first ablated by an agent when, on average, fewer than 3 MFU per crypt survive (Poisson distribution). These are presumably cells that are most resistant to the action of that particular agent. It has been argued from radiation studies, that approximately 80 MFU in a crypt of 160 proliferating cells share the survival characteristics of those final few that actually determine crypt survival (Potten and Hendry, 1983). In the cases of $HN_2$, ADR and 5-FU, it is possible that only 2 MFU were resistant to the action of the drugs, while the remaining 80 minus 2 (or 160 minus 2?) were profoundly sensitive. Against this interpretation are the facts that (a) such "sensitivity" was not expressed in a greatly increased number of necrotic cells relative to radiation (this paper), (b) experiments using graded low doses of drug plus high test doses of radiation failed to reveal a sensitive compartment (Moore, 1979; Moore & Broadbent, 1980), (c) the known cell cycle phase-specificities of the three drugs are quite different (e.g. Hill & Baserga, 1975), (d) while 5-FU has a very long biological half-life (Myers *et al.*, 1974) and might therefore kill many cells recruited from a more resistant state, $HN_2$ has a half-life of only a few minutes (Nadkarni *et al.*,

1956) and can be classed with radiation as an "acute" treatment. It may be that the response of a whole crypt cannot be interpreted simply in terms of the independent responses of cells of a particular, numerically-fixed population, but that considerations of cooperation within and between populations have a bearing on the observed overall result of a treatment. It could be speculated that for a microcolony to form, a focus (MFU) of more than one cell may be required, and that the number of cells per MFU may vary between cytotoxic agents. With regard to the number of necrotic cells *vs* MFU, Hendry & Potten (1982) considered whether the former might represent a sub-compartment of the latter. Plainly, if one adheres to the view that MFU are individual cells, this is not so for $HN_2$ (8 necrotic cells, 2MFU), a result which would then suggest that necrotic cells can arise from non-MFU. If the MFU compartment in CY- or ACT D-treated mice is also small, consisting of a very few cells that are highly resistant to these agents, then there is only a poor probability of detecting the response of such cells by histological means. The cell necrosis that is observed after CY or ACT D (Figures 3 and 4) would occur in a non-MFU, possibly amplification, compartment.

Certain other drugs that do not ablate crypts even with very large single doses, have a high value for the median of the distribution of numbers of necrotic cells *versus* cell position e.g. vincristine—median position 9 method (i) (Moore *et al.*, 1982) or hydroxyurea—median position 10 to 13 (Ijiri & Potten, 1983). The distribution curves for these drugs are more symmetric than those for radiation (median 5; Moore *et al.*, 1982) or ADR (median 5, this paper), not only because more cells are killed above position 14 but also because *fewer* cells are killed at or below position 5, the putative stem cell zone (Cheng & Bjerknes, 1980). We would now conclude, from results for CY and ACT D, that even a relatively high degree of histologically-defined cell death in this zone does not necessarily predispose to death of the crypt.

I am grateful to Mr D.A. Broadbent for technical assistance and Ms E. Mercer for typing the Ms. The work was supported by the Cancer Research Campaign (U.K.).

### References

AL-DEWACHI, H.S., WRIGHT, N.A., APPLETON, D.R. & WATSON, A.J. (1977). The effect of a single injection of hydroxyurea on cell population kinetics in the small bowel mucosa of the rat. *Cell Tissue Kinet.*, **10**, 203.

CHENG, H. & BJERKNES, M. (1980). The stem-cell zone of the mouse small-intestinal epithelium. In: *Cell Proliferation in the Gastrointestinal Tract* (Eds. Appleton *et al.*) Tunbridge Wells: Pitman Medical, p. 155.

GILBERT, C.W. (1974). A double minus log transformation of mortality probabilities. *Int. J. Radiat. Biol.*, **25**, 633.

HAGEMANN, R.F. (1980). The intestinal response to cytotoxic agents. In: *Cell Proliferation in the Gastrointestinal Tract* (Eds. Appleton *et al.*) Tunbridge Wells: Pitman Medical, p. 181.

HENDRY, J.H. & POTTEN, C.S. (1974). Cryptogenic cells and proliferative cells in intestinal epithelium. *Int. J. Radiat. Biol.*, **25**, 583.

HENDRY, J.H. & POTTEN, C.S. (1982). Intestinal cell radiosensitivity: A comparison for cell death assayed by apoptosis or by a loss of clonogenicity. *Int. J. Radiat. Biol.*, **42**, 621.

HENDRY, J.H., POTTEN, C.S., CHADWICK, C. & BIANCHI, M. (1982). Cell death (apoptosis) in the mouse small intestine after low doses: Effects of dose-rate, 14.7 MeV neutrons and 600 MeV (maximum energy) neutrons. *Int. J. Radiat. Biol.*, **42**, 611.

HILL, B.T. & BASERGA, R. (1975). The cell cycle and its significance for cancer treatment. *Cancer Treat. Rev.*, **2**, 159.

IJIRI, K. & POTTEN, C.S. (1983). Response of intestinal cells of differing topographical and hierarchical status to ten cytotoxic drugs and five sources of radiation. *Br. J. Cancer*, **47**, 175.

MOORE, J.V. (1979). Ablation of murine jejunal crypts by alkylating agents. *Br. J. Cancer*, **39**, 175.

MOORE, J.V. & BROADBENT, D.A. (1980). Survival of intestinal crypts after treatment by adriamycin alone or with radiation. *Br. J. Cancer*, **42**, 692.

MOORE, J.V. & HENDRY, J.H. (1978). Response of murine jejunal crypts to single doses of cyclophosphamide and radiation. *Int. J. Radiat. Oncol. Biol. Phys.*, **4**, 415.

MOORE, J.V., PEARSON, D. & DEAKIN, D.P. (1982). Gross and cellular response of intestinal crypts to single and fractionated doses of vincristine plus radiation: The influence of time between modalities. *Int. J. Radiat. Biol.*, **42**, 305.

MYERS, C.E., YOUNG, R.C., JOHNS, D.G. & CHABNER, B.A. (1974). Assay of 5-fluorodeoxyuridine 5′-monophosphate and deoxyuridine 5′-monophosphate pools following 5-fluorouracil. *Cancer Res.*, **34**, 2682.

NADKARNI, M.V., TRAMS, E.G. & SMITH, P.K. (1956). Observations on the rapid disappearance of radioactivity from blood after intravenous triethylenemelamine-$C^{14}$. *Proc. Am. Assoc. Cancer Res.*, **2**, 136.

POTTEN, C.S. (1977). Extreme sensitivity of some intestinal crypt cells to X- and γ-irradiation. *Nature*, **269**, 518.

POTTEN, C.S. (1980). Stem cells in small-intestinal crypts. In: *Cell Proliferation in the Gastrointestinal Tract.* (Eds. Appleton *et al.*) Tunbridge Wells: Pitman Medical, p. 141.

POTTEN, C.S. & HENDRY, J.H. (1983). Stem cells in murine small intestine In: *Stem Cells* (Ed. Potten) Edinburgh: Churchill-Livingstone, p. 155.

WITHERS, H.R. & ELKIND, M.M. (1970). Microcolony survival assay for cells of mouse intestinal mucosa exposed to radiation. *Int. J. Radiat. Biol.*, **17**, 261.

YAU, H.C. & CAIRNIE, A.B. (1979). Cell survival characteristics of intestinal stem cells and crypts of $\gamma$-irradiated mice. *Radiat. Res.*, **80**, 92.